# Pulmonary Tuberculosis, and Its Treatment by Hot Air

**Published:** 1889-05

**Authors:** 


### EDITORIAL.

### PULMONARY TUBERCULOSIS, AND ITS TREATMENT BY HOT AIR.

A method, proposed by Weigert, of treating tuberculosis by the inhalation of hot air has been attracting some attention lately. The plan is founded on the theory that tubercle-bacilli cannot live in a high temperature easily borne by man. "Even at a temperature of 38.5° C. [101.3° F.] they are very much weakened, and at a temperature greater than 42° C. [107.6° F.] they are killed." Man, however, can inhale, for a considerable time without injury, air as hot as 250° to 300° C. [482° to 572° F.] This heated air fills the lungs and, it is claimed, will raise the tissues to such a point as to kill the bacilli. The inhaling apparatus is a chamber heated by a lamp; two tubes pass off from the chamber, one from the top and the other from the bottom. The temperature is regulated by a thermometer. The patient takes the upper tube into the mouth and makes slow and full respirations, taking in as little air outside the tube as possible. The sittings are for one or two hours once every day.

The results claimed are increased expansion of the chest, in one case from 89 ctm. to 95½ ctm. within a few weeks. Dullness is cleared up, difficulty of breathing is diminished or disappears; the weight of the body is increased and the bacilli in the sputum diminish in number and show signs of a less vigorous growth.

The treatment has not been used long enough to determine whether or not this improvement in the patient is permanent.

The plan looks plausible enough at first to tempt to a careful trial. Still there are some reasons that lead us to doubt whether the hopes of the advocates of the treatment will ever be realized. As a general thing pathogenic bacteria in the tissues are hard to kill. Surgeons have learned by much sad experience that no amount of antiseptics will do away with the harm from dirty instruments or dirty hands. Obstetricians have learned to keep even clean hands off as much as possible. A suppurating wound cannot be cleaned by antiseptics alone—drainage is required, and that acts largely mechanically by carrying off the septic germs. Experience with all sorts of bacterial diseases has demonstrated that it is much better and easier to keep the germs out than to get them out after they have once got in. Tubercle-bacilli are no exception to the rule that bacteria in the tissues are hard to kill, as all know who have treated tuberculous ulcerations.

In tuberculosis the bacilli are imbedded in the lung tissue and in order to kill them this tissue must be heated to the fatal point of the bacteria and kept at this point for some little time. It is doubtful whether this occurs as the result of the treatment. It must be remembered that the air in the deepest inspiration penetrates only to the larger bronchial tubes. The mixture of this air with the residual air takes place slowly by the action of the cilia which line the tubes. While this is taking place the blood is circulating in great volume and with great speed in the lungs, all the blood in the body going through them in about half a minute. This great volume of blood must exert a powerful influence in keeping the temperature of the lungs the same as that of other parts of the body. It would seem that before any great elevation of temperature in the lungs could take place, all the blood must be heated nearly or quite as high as the lung tissue, consequently the temperature of the whole body must be raised. This does not occur. Those who have tried the treatment say that from .1° to .3° C. [.18° to .54° F.] higher than usual is all the elevation that the most careful examination ever shows. Such a rise would of course do the bacilli no harm.

Another thing should be borne in mind: In all ordinary cases of tuberculosis we have to do not with the tubercle-bacilli alone.

Early in the disease the tubercles soften and form cavities. These cavities in a soft and highly vascular tissue are filled with putrid pus which can not be drained off, and is constantly spreading over other portions of the lung tissue. This is absorbed, and causes more or less septic poisoning. Many if not the most of the worst symptoms of tuberculosis come from this secondary infection. The germs causing this putrefaction would be rather stimulated than injured by a high temperature.

Even if the treatment were to destroy the tubercle-bacilli, which we doubt, there would still remain the other infection, and the patient would be little if any better off than before.

We fear, therefore, that the improvement in the patients is only temporary; due, perhaps, to the practice of deep inspiration; and that the hopes of the advocates of the treatment are destined to disappointment.

